# Multikingdom oral microbiome interactions in early-onset cryptogenic ischemic stroke

**DOI:** 10.1093/ismeco/ycae088

**Published:** 2024-06-20

**Authors:** Muhammed Manzoor, Jaakko Leskelä, Milla Pietiäinen, Nicolas Martinez-Majander, Pauli Ylikotila, Eija Könönen, Teemu Niiranen, Leo Lahti, Juha Sinisalo, Jukka Putaala, Pirkko J Pussinen, Susanna Paju

**Affiliations:** Department of Oral and Maxillofacial Diseases, University of Helsinki, 00014 Helsinki, Finland; Department of Oral and Maxillofacial Diseases, University of Helsinki, 00014 Helsinki, Finland; Department of Oral and Maxillofacial Diseases, University of Helsinki, 00014 Helsinki, Finland; Industrial Biotechnology and Food Protein Production, VTT Technical Research Centre of Finland, 02044 Espoo, Finland; Department of Neurology, Helsinki University Hospital and University of Helsinki, 00290 Helsinki, Finland; Neurocenter, Turku University Hospital, University of Turku, 20521 Turku, Finland; Institute of Dentistry, University of Turku, 20500 Turku, Finland; Department of Public Health and Welfare, Finnish Institute for Health and Welfare, 00271 Helsinki, Finland; Department of Internal Medicine, Turku University Hospital and University of Turku, 20521 Turku, Finland; Department of Computing, University of Turku, 20500 Turku, Finland; Heart and Lung Center, Helsinki University Central Hospital, and Helsinki University, 00260 Helsinki, Finland; Department of Neurology, Helsinki University Hospital and University of Helsinki, 00290 Helsinki, Finland; Department of Oral and Maxillofacial Diseases, University of Helsinki, 00014 Helsinki, Finland; School of Medicine, Institute of Dentistry, University of Eastern Finland, 70211 Kuopio, Finland; Department of Oral and Maxillofacial Diseases, University of Helsinki, 00014 Helsinki, Finland

**Keywords:** metagenomics, oral microbiome, multikingdom, biomarkers, cerebrovascular disorders, stroke, cryptogenic ischemic stroke, saliva, co-abundance analysis

## Abstract

Although knowledge of the role of the oral microbiome in ischemic stroke is steadily increasing, little is known about the multikingdom microbiota interactions and their consequences. We enrolled participants from a prospective multicentre case–control study and investigated multikingdom microbiome differences using saliva metagenomic datasets (*n* = 308) from young patients diagnosed with cryptogenic ischemic stroke (CIS) and age- and sex-matched stroke-free controls. Differentially abundant taxa were identified using Analysis of Compositions of Microbiomes with Bias Correction (ANCOM-BC2). Functional potential was inferred using HUMANn3. Our findings revealed significant differences in the composition and functional capacity of the oral microbiota associated with CIS. We identified 51 microbial species, including 47 bacterial, 3 viral, and one fungal species associated with CIS in the adjusted model. Co-abundance network analysis highlighted a more intricate microbial network in CIS patients, indicating potential interactions and co-occurrence patterns among microbial species across kingdoms. The results of our metagenomic analysis reflect the complexity of the oral microbiome, with high diversity and multikingdom interactions, which may play a role in health and disease.

## Introduction

Stroke is a global health problem and the second-leading cause of death globally [1]. Ischemic stroke caused by arterial occlusion accounts for 87% of all strokes [2, 3]. Ischemic strokes are classified based on potential aetiologies [4], while they are commonly labelled as cryptogenic ischemic stroke (CIS) in the absence of a well-defined aetiology. The reported rates of CIS vary significantly (25%–70%) between populations [5–8]. Approximately 10%–15% of all strokes occur in adults aged 18–50 years [9]. The aetiology of ischemic stroke in young adults is different from that in older patients, and studies suggest that younger patients have an increased risk of death compared to stroke-free populations [10]. In young adults, the common modifiable risk factors for ischemic stroke are mostly behavioural, such as smoking, physical inactivity, obesity, diabetes, and hypertension [11, 12]. Additionally, emerging stroke risk factors like periodontitis are gradually being characterized, although evidence remains limited [13].

The microbial community inhabiting the oral cavity is known as the oral microbiota and is one of the most complex and dynamic microbial communities in the human body. In recent years, studies have elucidated the interplay between the oral microbiome and the development and progression of many oral and systemic diseases [14–16]. The diversity and composition of the oral microbiota have been linked to cardiovascular diseases, such as coronary artery disease, rheumatic heart disease, infective endocarditis, and congenital heart disease [17–22], indicating that the oral microbiota may play a role in their pathogenesis. The oral microbiota and its products (toxins, secreted proteins, or both) can easily spread to different body sites via the circulatory system, aspiration (via the lungs), or ingestion (to the gut), resulting in systemic inflammatory and immunologic responses [23].

There is growing evidence indicating that the microbiota plays a role in development of ischemic stroke, stroke prognosis, and recovery. Although several studies over the past decade have identified differences in the gut microbiome in ischemic stroke [24–27], its relationship with the oral microbiome is unclear. In a recent study, the oral microbiota composition and periodontitis-associated species within the genera *Porphyromonas*, and *Prevotella* from subgingival plaque samples were found to be associated with stroke [28]. In an animal experiment, mice receiving intragastric administration of salivary microbiota from periodontitis patients stimulated migration of interleukin-17A-producing cells from the gut to the brain and resulted in significantly poorer stroke outcomes [29]. Recently, the oral bacterial genera *Streptococcus*, *Prevotella*, *Veillonella*, and *Fusobacterium* from saliva samples were shown to be associated with ischemic stroke [30]. However, these studies on ischemic stroke tended to rely on small cohorts or data based on 16S rRNA gene sequencing. Therefore, greater genomic coverage, data output, and detection of a large number of bacterial and nonbacterial microbial communities, including archaea, viruses, and fungi are lacking [31]. In a recent prospective population-based shotgun metagenomic study, oral microbial subcommunities and multiple individual microbial taxa were associated with an increased risk of ischemic stroke in elderly Chinese women [32]. However, the complex relationships existing within oral microbiome have not been evaluated. Hence, there is a lack of comprehensive studies delivering an integrated and robust assessment of the association between CIS and oral microbiome.

We hypothesized that the multikingdom microbiota (bacteria, archaea, fungi, and viruses) of the oral cavity, along with its functional capacities, differ between patients with young-onset CIS and stroke-free controls. To explore this, we conducted shotgun metagenomic sequencing of saliva to identify microbiota and functional markers associated with CIS.

## Materials and methods

### Cohort description and patient involvement

Saliva samples were collected in the SECRETO Oral study, which is a substudy of the international, prospective, multicentre study SECRETO (Searching for Explanations for Cryptogenic Stroke in the Young: Revealing the Etiology, Triggers, and Outcome) (NCT01934725) that enrolled young adults (age 18–49 years) presenting with an imaging-positive first-ever acute CIS [33].

Patients were included after standardized diagnostic procedures, including brain magnetic resonance imaging, angiography of intracranial and extracranial vessels, echocardiography, and screening for coagulopathies. CIS was defined according to the atherosclerosis, small vessel disease, cardiac source, other cause (A-S-C-O) classification [34], with a few adaptations to reflect the true uncertainty in clinical practice in younger patients [33]. Age- (±5 years), sex-, and ethnicity-matched stroke-free community controls were sourced locally from each study centre. This involves various methods such as random searches through population registers, where feasible. Controls were included in a 1:1 ratio. The participants underwent a thorough structural interview at the time of recruitment. Participant education level and status of smoking were reported based on a questionnaire. Diabetes and hypertension data were collected from the medical records. Data on antibiotic use were collected from medical records. The modified Rankin Scale (mRS, cut-offs: mRS 0–1 vs. >1), a globally recognized stroke outcome measure, was used to evaluate the functional status of CIS patients at 3 months post-stroke [35]. Furthermore, the Hospital Anxiety and Depression Scale (HADS, cut-offs: depression score or anxiety score < 8 vs. ≥8) was used to measure anxiety and depressive symptoms in CIS patients [36].

### Clinical oral examination

Oral examinations for SECRETO Oral study were performed during April 2014 to February 2020 at the University of Helsinki and the University of Turku, Finland [37]. Clinical oral examination was performed in a standard dental office setting by the same periodontal specialist (SP) to avoid inter-examiner differences. Caries was diagnosed in the clinical setting based on the full International Caries Detection and Assessment System (ICDAS) code ranging from 4 to 6 (from a dark underlying shadow from dentin to an extensive distinct cavity with visible dentine) indicating the need for dental restoration [38]. The number of sites with probing pocket depth (PPD) of 4–5 mm and ≥6 mm and bleeding on probing (BOP) were recorded from six sites per tooth. Participants were divided into three categories based on the BOP score: healthy (BOP score ≥ 20%), localized gingivitis (BOP score 20%–33%), and generalized gingivitis (BOP score ≥ 33%) [39]. Periodontitis staging and grading were determined from panoramic tomographs according to the 2017 World Workshop on the Classification of Periodontal and Peri-Implant Diseases and Conditions [40].

### DNA extraction, metagenomic sequencing, and bioinformatics analysis

Sample processing, DNA extraction, metagenomic sequencing, and bioinformatics analysis are described in detail elsewhere [41]. Briefly, stimulated saliva samples were collected at the beginning of the oral examination into RNase/DNase-free tubes and stored at −70°C. Samples were thawed on ice, and 500 μl of saliva was mixed with 500 μl of lysis buffer (Chemagic DNA Blood 400-H96 Kit PerkinElmer) in MN type B bead tubes (Macherey-Nagel). Tubes were vortexed thoroughly and stored at −70°C until DNA extraction. The samples were thawed and 10 μl proteinase K was added to the mixture. Samples were lysed with a Bead Ruptor Elite Homogenizer (Omni, Inc) with 3 cycles of 1-min shaking (6 m/s) with a 5-min pause after each bead beating period. The samples were incubated at 65°C for 1 h and centrifuged at 8000 rpm for 1 min, and 6 μl of RNAse (10 mg/mL, Thermo Fisher Scientific) were added to 96 deep-well plates, and 600 μl of the above supernatant was added to the deep-well plates.

DNA extraction was performed with a ChemagicTM 360 instrument (PerkinElmer) using a Chemagic Saliva600 prefilled protocol and Chemagic DNA Blood 400-H96 Kit (PerkinElmer). DNA concentrations were measured using a DeNovix™ DS-11 Spectrophotometer/Fluorometer (DeNovix Inc, Wilmington) with the Qubit™ dsDNA Assay Kit (ThermoFisher). An Agilent TapeStation 4200 system was used for quality control of gDNA, and 260 ng (10 ng/μl) of DNA were used for the preparation of DNA libraries using NEBNext® Ultra™ II FS DNA Library preparation kit according to the manufacturer’s instructions.

Shotgun metagenomic paired-end sequencing (2 × 150 bp) was performed using an Illumina NovaSeq 6000 system. Quality control of the data was first performed using FastQC (v. 0.11.9) and MultiQC (v. 1.9). Most of the adapter sequences and adapters from the FASTQ were then removed. Quality trimming and remaining adapter remnants were removed using the Trimmomatic (v. 0.39) package [42]. Using the trimmed and filtered reads, host-associated sequences were removed via Kneaddata software (v. 0.10.0) (https://huttenhower.sph.harvard.edu/kneaddata/) with the human genome (GRCh38.p14) to generate clean fastq reads, resulting in an average of 3.67 ± 2.51 million paired-end reads per sample. The output was further processed with BBMap (v38.91) [43] to remove the duplicate reads. The remaining reads were then analyzed to obtain microbial taxonomy using Kraken2 (v. 2.1.2) [44], with default settings using prebuilt PlusPF database. Bracken (v. 2.7) was used to hone the species-level abundance estimates [45].

### Statistical analysis

Statistical analysis was performed with IBM SPSS Statistics software (v. 29.01.0; IBM, New York, USA). Variables were expressed as follows: continuous variables were presented as medians with interquartile range (IQR), while categorical variables were reported as counts with corresponding percentages. The differences in characteristics, and oral parameters between cases and controls were analyzed by Pearson χ^2^ test, Mann–Whitney test, and one-way ANOVA. All calculations and analyses of the microbiome data (including taxonomic and functional data and further visualizations) were performed using R statistical software package (v. 4.2.2). For microbiome analysis, we utilized specialized data containers in R designed for microbiome research, including packages such as phyloseq [46] and TreeSummarizedExperiment [47]. We removed taxonomic features with <10 sequence reads before analysis. Alpha diversity indices, such as the observed species index and Shannon index, were calculated using the R-package “vegan” (v. 2.6.4) [48]. Differences in beta diversity between cases and controls were assessed using the Permutational Analysis of Variance (PERMANOVA; function “adonis2”) from the R-package “vegan” (v. 2.6.4). To depict the diversity in taxonomic composition, we employed principal coordinate analysis (PCoA) based on the Bray-Curtis, Jaccard, weighted, and unweighted Unifrac distance metrics.

For differential abundance analysis, we first performed quality control filtering for taxonomic features and excluded microbial species that did not surpass the minimum prevalence (10% of samples). Composition and diversity analyses were reported based on this. Differential abundances of taxa (phylum and species) between study groups were identified using Analysis of Compositions of Microbiomes with Bias Correction (ANCOM-BC2) [49], incorporating covariates of age, smoking, educational status, hypertension, caries, and periodontitis. *P*-values were adjusted for the false discovery rate of multiple tests using the Benjamini-Hochberg method [50].

### Functional profiling

We further constructed functional profiles for each sample using the MetaCyc pathway database (https://metacyc.org/) using HUMAnN3 (v. 3.6), with default and the standard HuMAnN databases [51]. Further scripts embedded within HUMAnN3 (humann_rename_table and human_join_table) were used to align pathway abundances and coverage to merge all original output abundance tables into a single table using relative abundances data. Functional features significantly enriched in the metagenomes of either patients or controls were determined using the MaAsLin2 (v. 1.12.0) R package [52].

### Co-abundance analysis of the multikingdom oral microbiome

To explore single and multikingdom associations among species, we conducted a network analysis to establish microbial co-occurrence patterns in both the patients and control groups [53]. Correlation-based networks were constructed using the SparCC correlation method [54]. All microbial species meeting the 0.001 threshold were utilized in network construction, guided by a correlation coefficient threshold of 0.8 and a significance threshold of FDR-adjusted *P* < .05.

## Results

### Population characteristics

Table S1 provides summary data of the study participants and the main covariates. After applying exclusion criteria and accounting for occasional missing data, 155 samples from young-onset CIS and 153 age-and sex-matched samples from the control group were included in the analyses (Fig. S1). The median age (IQR) was similar between the case and control groups [patients, 41.81 (34.4–45.8) vs control, 42.10 (34.5–46.8); *P* = .457]; 58.8% were males. Over half of the participants had high education status (57.8% had postsecondary nontertiary education or tertiary education). There was a statistically significant difference in education level between cases and controls (*P* = .003). 44.2% of participants were smokers, and no significant differences were found between cases and controls (*P* = .123). The frequency of caries was higher in patients (45.8% vs 34.0%, *P* = .023). Periodontitis was observed in 26.5% of patients and 21.6% of controls. However, the difference in periodontal status between cases and controls was not significant (*P* = .169). Less than half (41.6%) of the patients had mRS values greater than 1 showing significant disability, and only 6.5% exhibited anxiety and depressive symptoms as measured using HADS (Table S1).

### Αlpha and beta diversity

**Figure 1:**
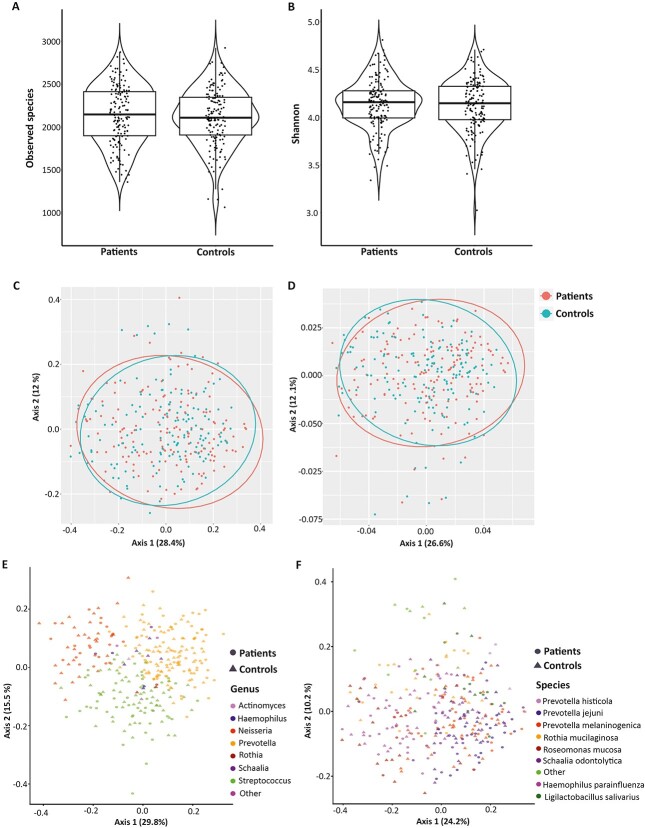
Oral microbiome diversity and characteristics; alpha diversity measurements in terms of (A) observed species and (B) Shannon index, and PCoA of beta diversity plots based on (C) Bray–Curtis dissimilarity and (D) weighted Unifrac distance metrics; PCoA ordination indicates sample similarity based on microbial taxonomic composition at the (E) genus and (F) species level; the colour indicates the most abundant taxa in each sample.

No differences were observed in alpha-diversity metrics between cases and controls (observed species; *P* = .440, Fig. 1A, Shannon; *P* = .860, Fig. 1B). However, we observed differences in alpha diversities among gender, caries, and smoking when analyzed separately for cases and controls (Table S2 and Fig. S2, see online supplementary material for a colour version of this figure).

Microbial community composition (beta diversity) did not differ between cases and controls (PERMANOVA, Bray-Curtis: *F* = 1.108, *P* = .325; Jaccard: *F* = 0.9490, *P* = .850; Weighted Unifrac: *F* = 1.0053, *P* = .403; Unweighted Unifrac: *F* = 0.9394, *P* = .837; Table S3). To further illustrate microbial community changes between cases and controls PCoA was performed using Bray-Curtis (Fig. 1C), Weighted Unifrac (Fig. 1D), Jaccard (Fig. S3 A, see online supplementary material for a colour version of this figure), and Unweighted Unifrac (Fig. S3B, see online supplementary material for a colour version of this figure) distances. Inter-individual variation in microbial composition was largely attributable to differences in the relative abundances of the most prevalent and abundant genera (Fig. 1E and Fig. S4, see online supplementary material for a colour version of this figure) and species (Fig. 1F).

### Community composition

**Figure 2:**
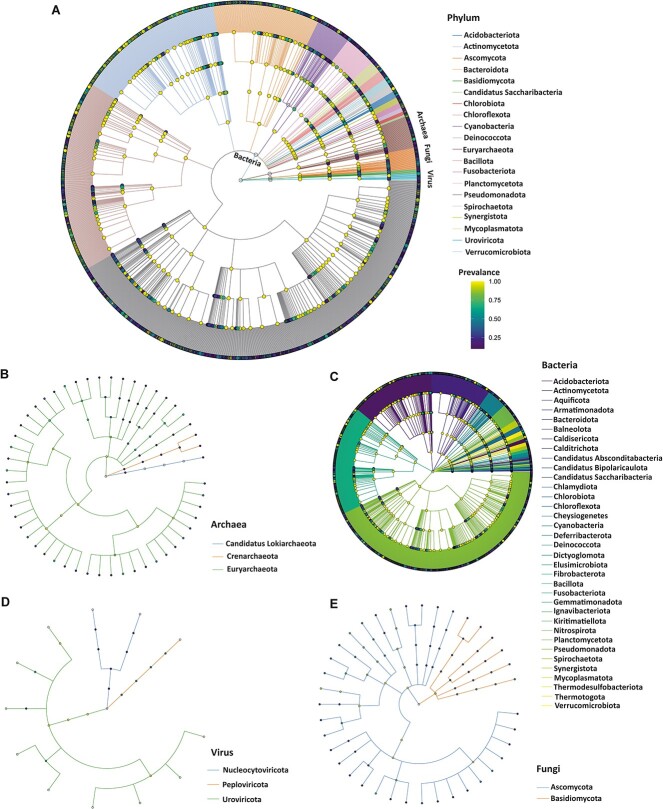
Metagenomics-based multikingdom taxonomic tree of the oral microbiota, including archaea, bacteria, viruses, and fungi; (A) taxonomic tree showing the prevalence of top phyla (*n* = 20) as judged by mean abundance in the saliva metagenome, and (B) taxonomic tree of archaea, and after prevalence filtering, the resulting saliva archaeaome consisted of 3 phyla and 93 species; (C) taxonomic tree of bacteria as judged by mean abundance; after prevalence filtering, the resulting saliva bacteriome consisted of 35 phyla and 5712 species; (D) taxonomic tree of viruses; the virome consisted of 3 phyla and 44 species; (E) taxonomic tree of fungi; saliva mycobiome consisted of 2 phyla and 62 species, and from the inner to outer circles, the taxonomic levels range from kingdom to species; the diameter of nodes indicates the abundance at different taxonomic levels.

After prevalence filtering (10%), the resulting microbiome data consisted of 43 phyla, and 5911 species. We constructed the taxonomic tree of the most abundant phyla to display the taxonomy composition and abundance (Fig. 2). A total of 5712 bacterial, 93 archaeal, 44 viral, and 62 fungal species were detected in samples meeting the prevalence thresholds (Table S4).

**Figure 3:**
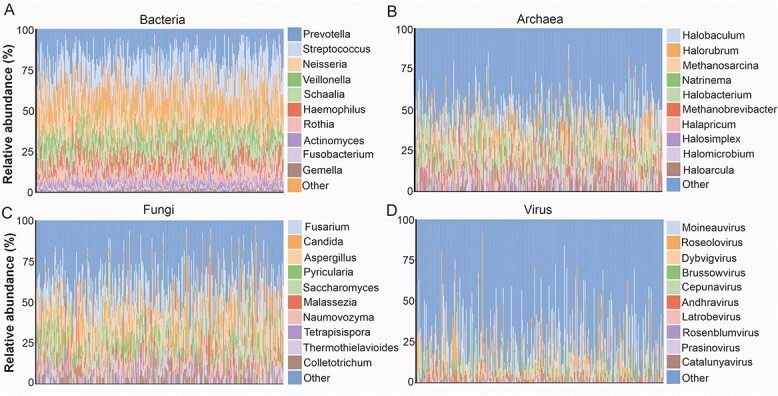
Taxonomic composition of metagenomes from saliva samples analyzed in this study; relative abundance of top 10 oral microbiome composition at the genus-level taxonomy for (A) bacteria, (B) archaea, (C) fungi, and (D) virus.

**Figure 4:**
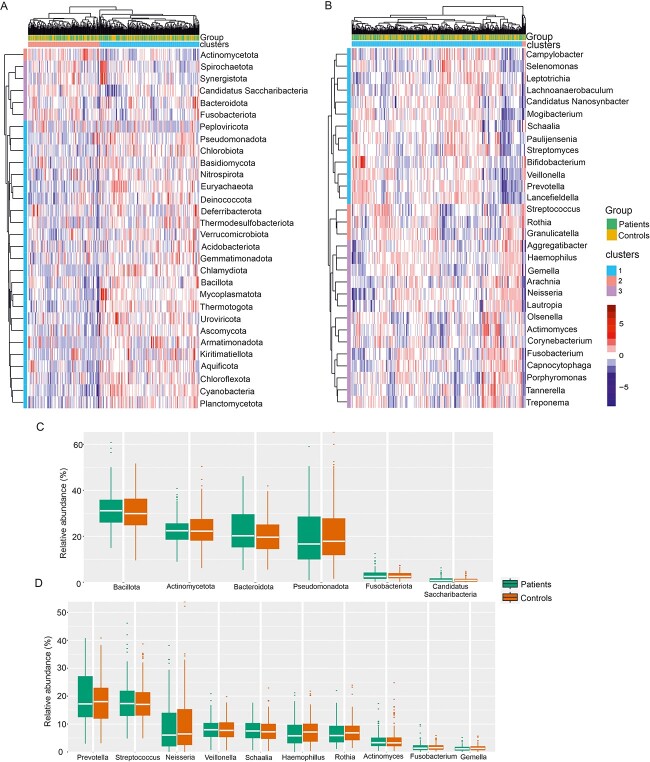
Differences in oral microbiota composition between patients and controls; cluster heatmap showing the proportions of taxa classified at the (A) phylum and (B) genus level between patients and controls; the top 30 most abundant taxa are shown; (C) relative abundance of top phyla in patients and controls; (D) relative abundance of top 10 genera in patients and controls.

The oral microbiome was dominated by the phyla Bacillota (Firmicutes), Actinomycetota (Actinobacteria), Bacteroidota (Bacteroidetes), Pseudomonadota (Proteobacteria), and Fusobacteriota (Fusobacteria), accounting on average for >98% of the saliva microbiota. In the kingdom bacteria, we identified 35 phyla, and 1579 genera. The most common bacterial genera present in saliva included *Prevotella*, *Streptococcus*, *Neisseria*, *Veillonella*, and *Schaalia* (Fig. 3A)*.* In the kingdom archaea, 3 phyla, 15 families, 49 genera, and 93 species were detected. The most common genera were *Halobaculum*, *Halorubrum*, *Methanosarcina*, *Natrinema*, and *Halobacterium* (Fig. 3B). In the kingdom fungi, 40 genera, and 62 species were detected. The most common fungal genera present in saliva included *Fusarium*, *Candida*, *Aspergillus*, *Pyricularia*, and *Saccharomyces* (Fig. 3C)*.* The viral metagenome consisted of 3 phyla, 15 genera, and 44 species. Among these 44 viral species identified, 22 (50%) were phages. The most common viral genera found in saliva included *Moineauvirus*, *Roseolovirus*, *Dybvigvirus*, *Brussowvirus*, and *Cepunavirus* (Fig. 3D)*.* While the predominant microbiota in the saliva was largely consistent among the patients and controls (Fig. 4 A and B), different relative abundances were observed when comparing the top 10 taxa at the phylum and genus levels (Fig. 4C and D).

### Multikingdom microbiome associated with CIS

ANCOM-BC2 analysis revealed multiple statistically significant differences between cases and controls, both in the crude model and in the model adjusted for age, educational status, hypertension, smoking, caries, and periodontitis. We identified 37 microbial species that were significant in the crude model, and 51 species that were significant in the adjusted model. At the phylum level, only Candidatus Absconditabacteria associated positively with controls in the crude model (*P* < .05) (Table S5). At the species level, many oral taxa clearly exhibited higher abundances in the cases than in the controls. In the adjusted model, the bacterial species that showed the highest increase in abundance in patients were *Brenneria goodwinii, Variovorax boronicumulans, Pseudomonas* sp. AN-B15, *Actinoalloteichus sp.* GBA129-24, and *Thiomonas arsenitoxydans* (*P* < .05). Conversely, the bacterial species *Metamycoplasma alkalescens*, *Streptomyces* sp. LBUM 1475, *Pseudonocardia dioxanivorans*, *Arthrobacter dokdonella*, and *Francisella frigiditurris* were less abundant in cases than in controls (*P* < .05).

Furthermore, we found that three viral and one fungal species were associated with CIS. The abundance of the viral species *Dybvigvirus crAssphage* cr131_1 was higher in the cases than in the controls (*P* < .05). Conversely, the abundances of the viral species *Brussowvirus* ALQ132 and *Moineauvirus* Abc2 were lower in patients than in controls (*P* < .05). Additionally, *Fusarium venenatum* was the significant fungal species among the controls (*P* < .05) (Table S5).

### Microbial functional differences in CIS

We identified five metagenomic pathways whose abundance across patients and controls was statistically significantly different (*P* < .05, *q* < 0.25) (Table S6).The patients had an increased abundance of pathways associated with inosine 5′ phosphate biosynthesis II and III (PWY-6124 and PWY-7234) and stachyose degradation (PWY-6527), while controls had an increased abundance of pathways associated with 8-amino 7-oxononanoate biosynthesis I (PWY-6519) and tetrapyrrole biosynthesis II (from glycine) (PWY-5189).

### Differences in the multikingdom co-abundance network between patients and controls

**Figure 5:**
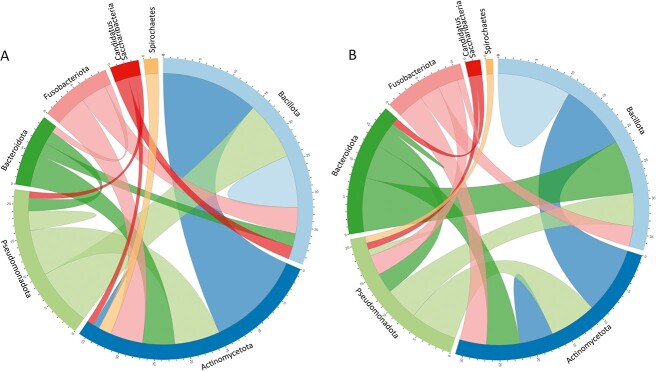
The Circos plot illustrates correlations between different microbial taxa presented at the phylum level of taxonomy (correlation cut-off at *r* ≥ 0.8) in both (A) patients and (B) control samples; the number of linkages from one phylum to another or within the same phylum is depicted; the size of the bars within each phylum indicates the relative abundance levels in the samples.

To investigate the potential interplay of species between kingdoms and their role in CIS, we conducted a co-abundance association analysis. The ecological network within patients appeared notably more intricate than that within the controls: patients’ microbiome network displayed 1043 features with 58 significant peripheral node modules, while the corresponding number of associations was 1036 features and 56 significant peripheral node modules in controls (Table. S7). This complexity indicated higher significant correlations and increased species clustering in patients compared to controls. Circos plots showed strong (*r* ≥ 0.8) correlations among multiple bacterial phyla, depicting the linkages between different phyla or within the same phylum (Fig. 5).

## Discussion

Utilizing this well-characterized cohort of young CIS patients and their age- and sex-matched stroke-free controls, we observed that CIS is associated with multikingdom perturbations of the oral microbiome. We identified a series of oral markers, including 47 bacterial, 3 viral, and one fungal species associated with CIS. Co-abundance analysis of the ecological network in CIS patients revealed positive associations across kingdoms. Our findings show that the diversity and composition of the oral microbiome and its multikingdom interactions may play a role in CIS.

We observed that saliva microbial diversity (alpha and beta) between the two groups was unchanged. This is consistent with another study that used 16S rRNA amplicon sequencing and compared saliva and subgingival samples of stroke patients, patients developing stroke-associated pneumonia, and patients with stroke-like symptoms who were not diagnosed with stroke [28]. In a recent prospective shotgun metagenomic study, alpha and beta diversity of the oral microbiome were associated with the subsequent risk of ischemic stroke [32]. These differences between studies may be due to age (>20 years difference in the mean age), ethnic or geographic differences of subjects, saliva sampling methods, nucleic acid extraction methods, or the bias of sequencing methods. In the present study, the alpha diversity of saliva microbiota in CIS was associated (Shannon index) with gender, smoking, and antibiotic use. In previous studies, systemic antibiotics prescribed in any clinical indications were associated with the saliva microbiome for 5–8 months in adults [55] and even for years among children [56].

We found that the predominant microbiota in saliva was largely consistent between cases and controls. The most abundant genera in the oral cavity are *Streptococcus*, *Haemophilus*, *Prevotella*, and *Veillonella* [57]. In the present study, *Prevotella* was more abundant than *Streptococcus.* At the species level, *B. goodwinii, V. boronicumulans, Pseudomonas* sp. AN-B15, *Actinoalloteichus sp.* GBA129–24, and *T. arsenitoxydans* were the most significant bacterial species in cases, while *Mycoplasma alkalescens*, *Streptomyces* sp. LBUM 1475, *P. dioxanivorans*, *A. dokdonella*, and *F. frigiditurris* were the most significant in controls. Our findings suggest that these bacteria may be involved in the pathogenic processes before or after CIS, but their role in stroke risk or stroke prognosis remains unknown.

Another novel finding of our study is the clear differences in nonbacterial communities in patients compared to controls. Oral archaea, fungi, and viruses are far less numerous and diverse than oral bacteria and have remained relatively understudied [58]. Previous studies have shown that a healthy oral cavity is home to at least 75 fungal genera. The most abundant genera were *Candida*, *Cladosporium*, *Aureobasidium*, *Aspergillus*, *Fusarium*, *Cryptococcus*, and *Saccharomycetales* [59]*.* Similarly, in the present study, we found 40 fungal genera, of which *Candida, Aspergillus, Fusarium*, and *Saccharomyces* were among the most common. *F. venenatum* was the only fungal species that differed between patients and controls. *F. venenatum*, a species of filamentous fungus belonging to the phylum Ascomycota and genus *Fusarium*, is a microfungus known for its high protein content [60]. Nevertheless, our results indicate that shotgun metagenome sequencing of saliva is a useful tool for investigating the oral mycobiome.

The oral cavity is home to a diverse array of viruses collectively called the oral virome, including bacteriophages (viruses that infect bacteria), eukaryotic viruses (viruses that infect eukaryotic cells; such as those found in humans), and archaeal viruses (viruses that infect archaea, a domain of single-celled microorganisms). Similar to the bacteriome, the oral virome plays a significant role in oral health and disease [61]. The most common viral genera found in the saliva were *Moineauvirus, Roseolovirus, Dybvigvirus, Brussowvirus*, and *Cepunavirus*. In the present study, we observed that the oral virome of CIS patients was dominated by *Dybvigvirus crAssphage* cr131_1 whereas the species *Brussowvirus* ALQ132 and *Moineauvirus* Abc2 were less prominent. All these significant species belong to the phylum Uroviricota, and viruses from this phylum primarily infect bacteria. Previous studies have suggested that bacteriophages from the phylum Uroviricota can influence the composition and dynamics of bacterial communities in the gut microbiota [62]. Phage communities within the oral microbiome exhibit considerable biogeographic diversity. Among the phages identified in this study, *Streptococcus* phage was the predominant one. *Streptococcus* phage, also known as a bacteriophage specific to *Streptococcus* bacteria, is a type of virus that infects and replicates within bacteria of the *Streptococcus* genus. However, because of the scarcity of experimentally characterized phages and the lack of readily available model systems, the ecological and physiological importance of phages in the oral microbiome, as well as their influence on host–microbe interactions, remain uncertain. Moreover, the viruses in saliva may act as hosts for pathogenic genes and their function in the oral environment [63]. Despite the unknown role of nonbacterial communities in saliva or their association with CIS, our study can be considered an opening for further basic or clinical research.

A recent analysis of the oral microbiome network, which reveals its inherent complexity involving associations across multiple kingdoms [64], aligns with our observations. The ecological network observed among patients displayed higher complexity with increased module hubs and peripheral node associations than the control group, revealing emergent interactions within and between kingdoms in the oral microbiome. From the CIS perspective, these interactions can be of concern, but their impact on general health remains largely unexplored.

We identified five metagenomic pathways whose differential abundance across patients and controls was significant. CIS patients had an increased abundance of pathways associated with inosine 5′ phosphate biosynthesis II and III and stachyose degradation. Inosine monophosphate plays a central role in intracellular purine metabolism and can serve as an extracellular signalling molecule regulating inflammation [65]. Stachyose is a functional oligosaccharide and can facilitate the growth of the dominant bacteria in the gut [66]. These results suggest that the oral microbiome may play a role in communication within the oral-gut-brain axis.

Multiple methods have been used to identify the differentially abundant microbes in microbiome studies. Many tools identify vastly different numbers and sets of significant operational taxonomic units, and the results depend on data preprocessing [67]. Among these methods, ANCOM-BC2 incorporates bias correction techniques to improve accuracy and enhance the detection of features by reducing false positives and false negatives, leading to more robust and interpretable results by outputting the log-fold change difference for the taxa between groups.

We acknowledge the following strengths and limitations. The strengths of our study included the prospective design, the age-sex balanced subset assessment, and detailed phenotyping of oral and periodontal status. In addition, the oral examination was performed by the same periodontal specialist to avoid inter-examiner differences. Moreover, utilization of shotgun metagenomic sequencing rather than 16S rRNA amplicon sequencing enabled greater genomic coverage and data output [31], thereby facilitating the detection of a large number of multikingdom species and increasing the prediction of pathways. Limitations of the study include the fact that even though our study was a multicentre case–control study, we only included patients treated in two centres, which may potentially limit the generalizability of the findings. Participant education level and smoking status were self-reported introducing a possibility for bias. As the saliva samples were collected 8–12 weeks postevent, the composition of oral microbiome may have been affected by the event itself or the stroke medications. Another important point to note is that oral hygiene practices or plaque amounts were not assessed among the participants. These factors could potentially contribute to oral microbiome dysbiosis [68]. Moreover, the probiotic supplementation status of patients is unknown. Probiotics have demonstrated oral health benefits by influencing the microbiome and host [69]. Finally, we identified some members of the oral microbiota that could be used as indicators of disease status. These microbes in the saliva are usually swallowed and transmitted to the gut, where they may induce neuroinflammation via their metabolites [70]. However, it was not possible to elucidate the mechanisms in this study. Thus, further work is needed to understand how the microbiota and its metabolism shape the “oral-gut-heart-brain” axis and the putative mechanisms linking them to stroke.

## Conclusions

We identified a range of oral markers including bacterial, fungal, and viral species associated with CIS in young individuals, highlighting the complexity of the microbiome contributing to the homeostatic oral environment. Our findings emphasize the significance of integrated multikingdom analyses for identifying microbial features associated with CIS. The association between CIS and the multikingdom microbiome may elucidate the role of the oral microbiome in stroke pathology.

## Supplementary Material

Supplemental_Material_ycae088_Fig_S1

Supplemental_Material_ycae088_Fig_S2

Supplemental_Material_ycae088_Fig_S3

Supplemental_Material_ycae088_Fig_S4

Supplemental_Material_ycae088_Table_S1

Supplemental_Material_ycae088_Table_S2

Supplemental_Material_ycae088_Table_S3

Supplemental_Material_ycae088_Table_S4

Supplemental_Material_ycae088_Table_S5

Supplemental_Material_ycae088_Table_S6

Supplemental_Material_ycae088_Table_S7

## Data Availability

The metagenomic data are available from the European Genome-Phenome Archive (accession number: EGAS00001007505). The source code for the analyses is available at https://zenodo.org/doi/10.5281/zenodo.10972459
