# Supplementary material for: Multikingdom oral microbiome interactions in early-onset cryptogenic ischemic stroke
Source: ISME Commun. 2024 Jun 20;4(1):ycae088. doi: 10.1093/ismeco/ycae088 (PMC11235082; doi:10.1093/ismeco/ycae088)
Supplement: Supplemental_Material_ycae088_Fig_S1 [file supplemental_material_ycae088_fig_s1.pdf]

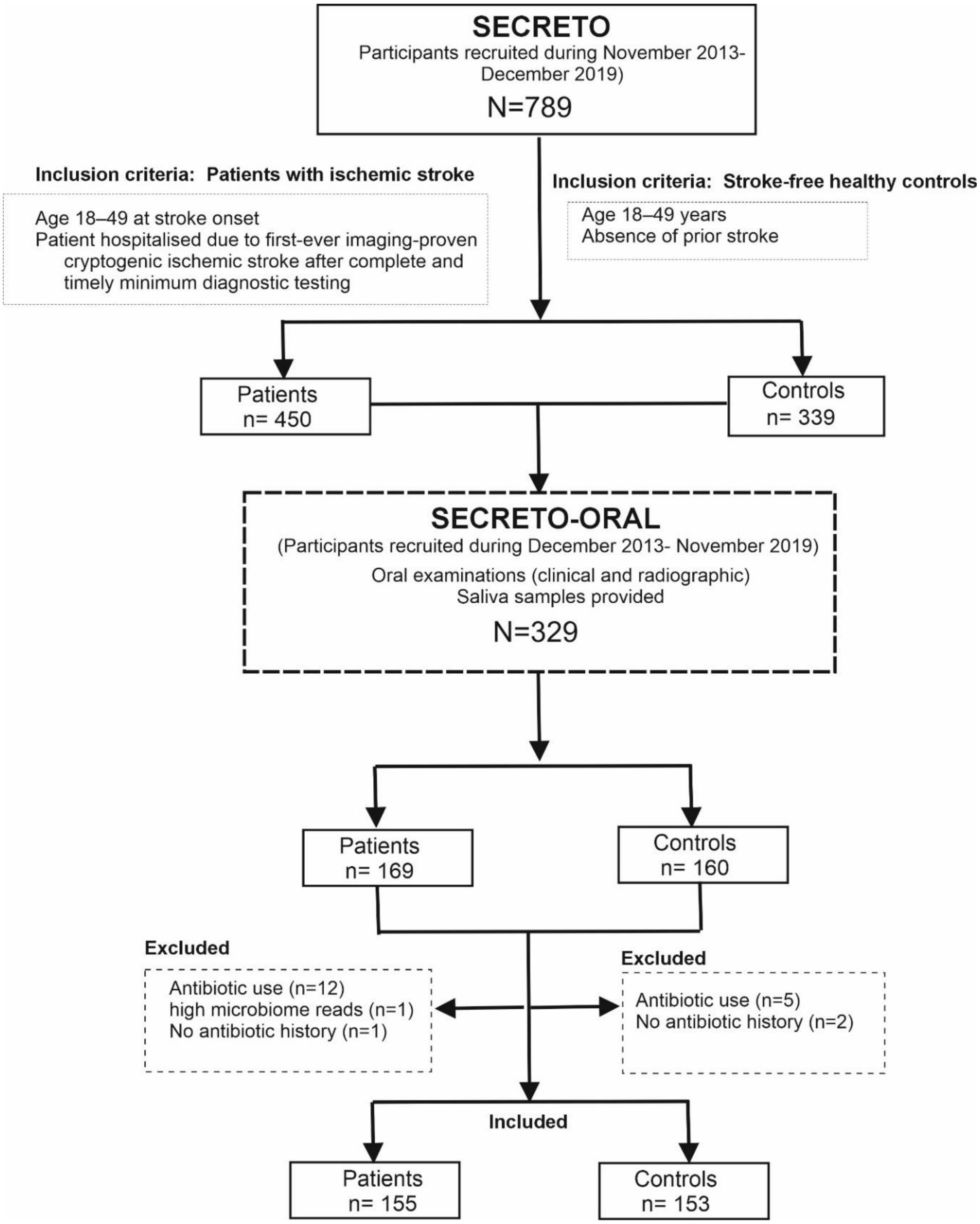

Fig. S1. Flowchart describing cryptogenic ischemic stroke patients and controls recruited for the study. Participants recruited during 2013–2019 who entered the SECRETO Oral study, including 169 cases and 160 controls. After exclusions, a total of 308 eligible cases were included in the present study, where the oral microbiome composition was analysed using shotgun metagenomics sequencing.
