# Supplementary material for: Multikingdom oral microbiome interactions in early-onset cryptogenic ischemic stroke
Source: ISME Commun. 2024 Jun 20;4(1):ycae088. doi: 10.1093/ismeco/ycae088 (PMC11235082; doi:10.1093/ismeco/ycae088)
Supplement: Supplemental_Material_ycae088_Fig_S2 [file supplemental_material_ycae088_fig_s2.pdf]

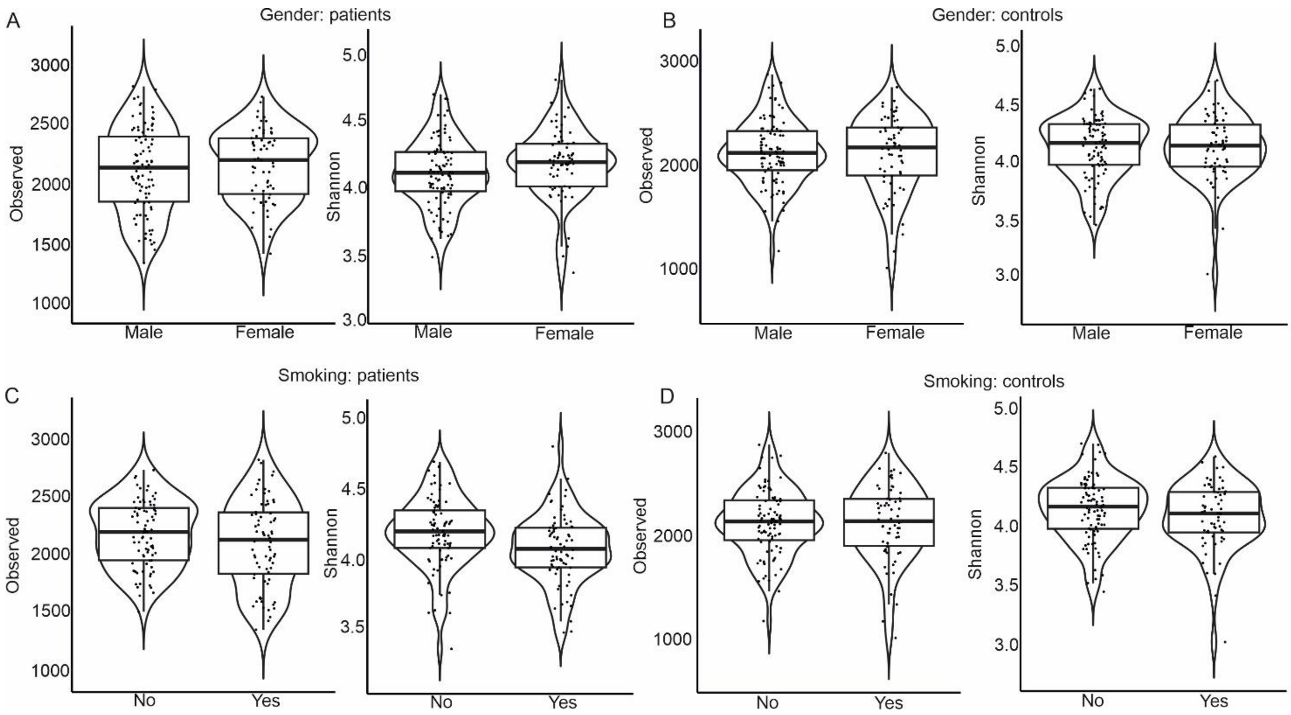

Fig. S2. Box plot illustrating the alpha diversity, measured by the observed species and Shannon index, separately for the patients and control groups. (A) Alpha diversity by gender in the patients group. (B) Alpha diversity by gender in the control group. (C) Alpha diversity by smoking status in the patients group. (D) Alpha diversity by smoking status in the control group.
