# Supplementary material for: Multikingdom oral microbiome interactions in early-onset cryptogenic ischemic stroke
Source: ISME Commun. 2024 Jun 20;4(1):ycae088. doi: 10.1093/ismeco/ycae088 (PMC11235082; doi:10.1093/ismeco/ycae088)
Supplement: Supplemental_Material_ycae088_Fig_S3 [file supplemental_material_ycae088_fig_s3.pdf]

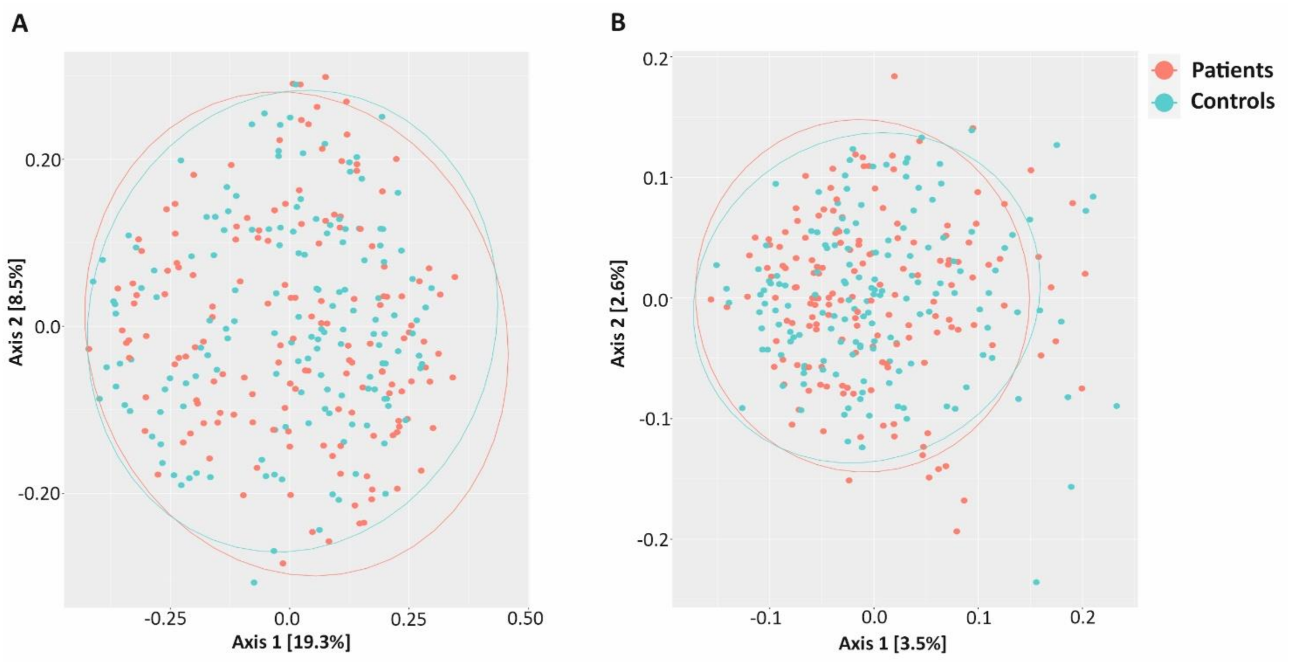


**Fig. S3.** Principal coordinate analysis (PCoA) of beta diversity plots based on (A) Jaccard and (B) Unweighted UniFrac distance metrics between patients and controls. The percentages in brackets indicate the proportion of variation explained by each PCoA axis.
