# Supplementary material for: Multikingdom oral microbiome interactions in early-onset cryptogenic ischemic stroke
Source: ISME Commun. 2024 Jun 20;4(1):ycae088. doi: 10.1093/ismeco/ycae088 (PMC11235082; doi:10.1093/ismeco/ycae088)
Supplement: Supplemental_Material_ycae088_Fig_S4 [file supplemental_material_ycae088_fig_s4.pdf]

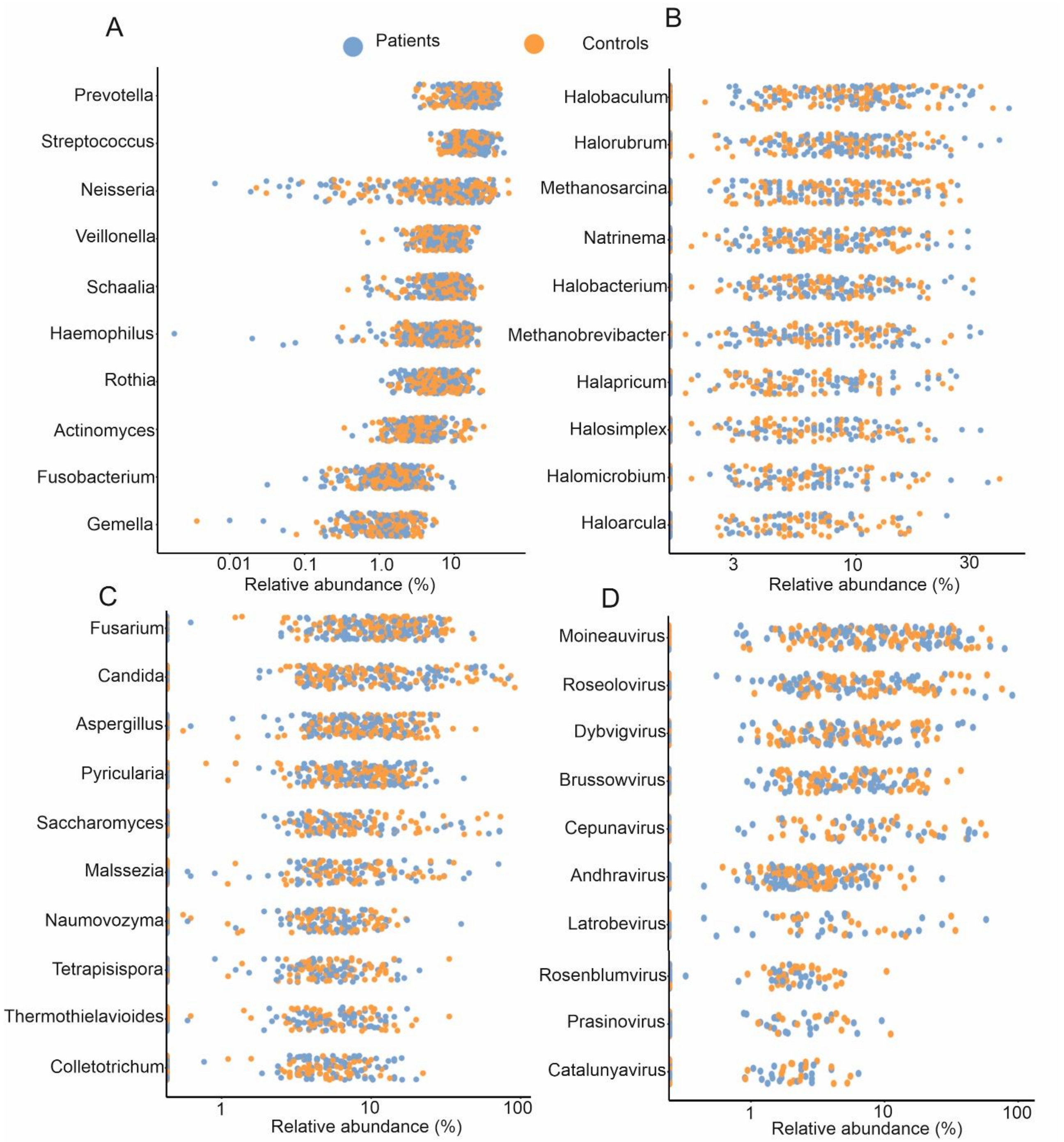

Fig. S4. Relative abundances of the 10 most abundant genera that were detected at 10% prevalence in the study participants. (A) Bacteria, (B) Archaea, (C) Fungi, and (d) Virus. Each dot represents one individual, and the darker regions indicate more populated areas of the abundance landscape.
