# Supplementary material for: Multikingdom oral microbiome interactions in early-onset cryptogenic ischemic stroke
Source: ISME Commun. 2024 Jun 20;4(1):ycae088. doi: 10.1093/ismeco/ycae088 (PMC11235082; doi:10.1093/ismeco/ycae088)
Supplement: Supplemental_Material_ycae088_Table_S1 [file supplemental_material_ycae088_table_s1.pdf]

Table S1. Characteristics of patients with cryptogenic ischemic stroke and stroke-free controls included in the study**.**

		Patients (n=155)	Controls (n=153)	*p*-value
Gender	Male (%)	90 (58.1)	91 (59.5)	0.446*
	Female (%)	65 (41.9)	62 (40.5)	
Age (median), years		41.81 (34.4-45.8)	42.10 (34.5-46.8)	0.457#
Education	primary or lower and upper secondary education	78 (50.3)	51 (34.2)	**0.003***
	post-secondary non-tertiary education or tertiary education	77 (49.7)	98 (65.8)	
BMI, kg/m^2		26.29 (23.7-29.7)	25.82 (23.7-28.9)	0.417#
Smoking, ever (%)		74 (47.7)	62 (40.5)	0.123*
Hypertension, yes (%)		38 (24.5)	26 (17.0)	0.068*
Diabetes diagnosis (%)		6 (3.9)	6 (3.9)	0.606*
Antibiotics (preceding 3 months), yes (%)		8 (5.2)	7 (4.6)	0.510*
Periodontal status	Healthy	10 (6.5)	7 (4.6)	0.169¤
	Localized gingivitis	27 (17.4)	46 (30.5)	
	Generalized gingivitis	73 (47.1)	65 (43.0)	
	Periodontitis, yes (%)	41 (26.5)	33 (21.6)	
BOP (%), median		41.0 (31.0-50.6)	35.7 (26.3-44.2)	0.525
Caries, yes (%)		71 (45.8)	52 (34.0)	**0.023***
Modified Rankin Scale (mRS)	0-1	90 (58.4)	-	-
	>1	64 (41.6)	-	
Hospital Anxiety and Depression Scale (HADS) score	<8	143 (93.5)	-	-
	≥8	10 (6.5)		

*χ^2 test; #Mann-Whitney U test; ¤ One-way ANOVA; Significant p-values are bolded.
Missing information: education 4; periodontitis 6; mRS 2; HADS 1.
