# Supplementary material for: Multikingdom oral microbiome interactions in early-onset cryptogenic ischemic stroke
Source: ISME Commun. 2024 Jun 20;4(1):ycae088. doi: 10.1093/ismeco/ycae088 (PMC11235082; doi:10.1093/ismeco/ycae088)
Supplement: Supplemental_Material_ycae088_Table_S2 [file supplemental_material_ycae088_table_s2.pdf]

Table S2. Alpha diversity, measured in terms of the observed species and Shannon indices separately, in both the patients with cryptogenic ischemic stroke and stroke-free controls.

	Observed	Shannon
**Patients**		
gender	0.550	**0.047**
caries	0.210	0.720
smoking	0.170	**0.0014**
antibiotics use (preceding 1–3 months)	0.230	**0.05**
**Controls**		
gender	0.700	0.970
caries	0.580	**0.037**
smoking	0.860	0.210
antibiotics use (preceding 1–3 months)	0.620	0.200
