# Supplementary material for: Multikingdom oral microbiome interactions in early-onset cryptogenic ischemic stroke
Source: ISME Commun. 2024 Jun 20;4(1):ycae088. doi: 10.1093/ismeco/ycae088 (PMC11235082; doi:10.1093/ismeco/ycae088)
Supplement: Supplemental_Material_ycae088_Table_S3 [file supplemental_material_ycae088_table_s3.pdf]

Table S3. Dissimilarity tests of microbial community composition between cryptogenic ischemic stroke patientsand controls using Permutational Multivariate Analysis of Variance (PERMANOVA) based on Bray-Curtis, Jaccard, Weighted and Unweighted Unifrac distance metrics. PERMANOVA: permutational multivariate analysis of variance.

	SumOfSqs	R^2	F-value	*p*-value
**Bray-Curtis**	0.1121	0.0036	1.1089	0.325
**Weighted Unifrac**	0.0032	0.0032	1.0053	0.403
**Unweighted Unifrac**	0.1588	0.0031	0.9394	0.837
**Jaccard**	0.1995	0.0030	0.9490	0.850
