# Supplementary material for: Multikingdom oral microbiome interactions in early-onset cryptogenic ischemic stroke
Source: ISME Commun. 2024 Jun 20;4(1):ycae088. doi: 10.1093/ismeco/ycae088 (PMC11235082; doi:10.1093/ismeco/ycae088)
Supplement: Supplemental_Material_ycae088_Table_S4 [file supplemental_material_ycae088_table_s4.pdf]

Table S4. The distribution of the total number of reads after processing and the taxa of the oral core microbiome were analyzed using shotgun metagenomics sequencing.

Number of raw reads after processing	33, 956, 066				
Average number of reads	110, 246				
**Taxonomy**	N (10% prevalence)	Bacteria	Archaea	Fungi	Virus
**Phylum**	43	35	3	2	3
**Class**	98	78	7	10	3
**Order**	213	184	12	14	3
**Family**	478	433	15	23	7
**Genus**	1683	1579	49	40	15
**Species**	5911	5712	93	62	44
