# Supplementary material for: Multikingdom oral microbiome interactions in early-onset cryptogenic ischemic stroke
Source: ISME Commun. 2024 Jun 20;4(1):ycae088. doi: 10.1093/ismeco/ycae088 (PMC11235082; doi:10.1093/ismeco/ycae088)
Supplement: Supplemental_Material_ycae088_Table_S5 [file supplemental_material_ycae088_table_s5.pdf]

Table S5. Differential abundance analysis using ANCOM-BC2 revealed significant differences at the phylum and species levels between cases and controls, both in the unadjusted model and in the model adjusted for age, educational status, hypertension, smoking, caries, and periodontitis.

**Phylum crude adjusted**						
**No**	**taxon**	**Enriched group**	**lfc_Group_patient**	**se_Group_patient**	**W_Group_patient**	**q-values**
1	Candidatus Absconditabacteria	controls	-0.377	0.053	-7.103	0.000
**Crude model species**						
**No**	**taxon**	**Enriched group**	**lfc_Grouppatient**	**se_Grouppatient**	**W_Grouppatient**	**q-values**
1	Metamycoplasma hyosynoviae	patients	0.463	0.059	7.881	7.20E-07
2	Metamycoplasma alkalescens	patients	0.423	0.052	8.127	8.47E-06
3	Candidatus Absconditicoccus praedator	controls	-0.377	0.053	-7.152	8.86E-06
4	Variovorax boronicumulans	controls	-0.366	0.055	-6.689	3.22E-05
5	Pseudomonas sp. AN-B15	controls	-0.383	0.051	-7.542	6.68E-05
6	Actinoalloteichus sp. GBA129-24	controls	-0.355	0.053	-6.760	0.000
7	Veillonella sp. OK1	patients	0.524	0.087	6.022	0.000
8	Streptomyces sp. KPB2	controls	-0.368	0.052	-7.073	0.000
9	Mahella australiensis	controls	-0.305	0.051	-5.919	0.001
10	Hydrogenophaga crassostreae	controls	-0.342	0.051	-6.673	0.001
11	Xanthobacter autotrophicus	controls	-0.318	0.051	-6.246	0.002
12	Pseudonocardia dioxanivorans	patients	0.302	0.056	5.417	0.002
13	Burkholderia sp. MS455	patients	0.331	0.051	6.459	0.002
14	Novosphingobium sp. EMRT-2	controls	-0.295	0.052	-5.714	0.003
15	Microbacterium sp. SGAir0570	controls	-0.326	0.054	-6.051	0.004
16	Amphritea japonica	controls	-0.281	0.052	-5.369	0.006
17	Levilactobacillus zymae	patients	0.282	0.051	5.510	0.008

18	Brucella anthropi	controls	-0.347	0.067	-5.145	0.010
19	Bacillus sp. FJAT-14266	controls	-0.286	0.054	-5.263	0.010
20	Paenibacillus kribbensis	controls	-0.301	0.052	-5.839	0.010
21	Rhodococcus sp. WAY2	controls	-0.272	0.053	-5.143	0.011
22	Arthrobacter dokdonella	patients	0.281	0.052	5.426	0.012
23	Pseudomonas sp. BIOMIG1BAC	controls	-0.299	0.053	-5.630	0.013
24	Brenneria goodwinii	controls	-0.282	0.053	-5.349	0.014
25	Polymorphobacter sp. PAMC 29334	controls	-0.279	0.051	-5.458	0.020
26	Plantactinospora sp. BB1	controls	-0.271	0.054	-4.969	0.021
27	Hydrogenophaga sp. PBL-H3	controls	-0.265	0.053	-5.004	0.025
28	Stenotrophomonas sp. CW117	controls	-0.306	0.060	-5.084	0.026
29	Exiguobacterium sp. MH3	patients	0.288	0.052	5.584	0.026
30	Microvirga ossetica	patients	0.257	0.052	4.962	0.028
31	Sphingobacterium sp. B2	controls	-0.278	0.051	-5.419	0.035
32	Streptomyces sp. WAC00303	controls	-0.254	0.052	-4.907	0.036
33	Geminocystis sp. NIES-3708	controls	-0.262	0.050	-5.208	0.038
34	Metamycoplasma phocicerebrale	patients	0.272	0.053	5.150	0.041
35	Streptomyces sp. BSE6.1	patients	0.284	0.052	5.418	0.047
36	Corynebacterium ceti	patients	0.255	0.051	4.980	0.047
37	Sulfuriferula plumbiphila	patients	0.256	0.051	5.012	0.048
**Species adjusted model for age, educational status, hypertension, smoking, caries, and periodontitis**						
**No**	**taxon**	**Enriched group**	**lfc_Grouppatient**	**se_Grouppatient**	**W_Grouppatient**	**q-values**
1	Brussowvirus ALQ132	controls	-0.534	0.056	-9.457	2.84E-07
2	Brenneria goodwinii	controls	-0.476	0.053	-9.038	3.11E-07
3	Metamycoplasma alkalescens	patients	0.508	0.053	9.506	2.47E-06
4	Variovorax boronicumulans	controls	-0.388	0.056	-6.902	2.35E-05
5	Pseudomonas sp. AN-B15	controls	-0.444	0.052	-8.511	3.22E-05
6	Actinoalloteichus sp. GBA129-24	controls	-0.429	0.054	-7.961	5.24E-05
7	Streptomyces sp. LBUM 1475	patients	0.444	0.057	7.840	5.63E-05

8	Pseudonocardia dioxanivorans	patients	0.348	0.056	6.171	6.44E-05
9	Thiomonas arsenitoxydans	controls	-0.380	0.053	-7.210	0.000
10	Arthrobacter dokdonella	patients	0.378	0.054	7.063	0.000
11	Frankia sp. QA3	controls	-0.350	0.056	-6.298	0.000
12	Novosphingobium sp. EMRT-2	controls	-0.362	0.053	-6.771	0.000
13	Francisella frigiditurris	patients	0.380	0.053	7.219	0.000
14	Streptomyces sp. KPB2	controls	-0.429	0.053	-8.069	0.000
15	Nostoc sp. NIES-4103	patients	0.342	0.052	6.582	0.000
16	Metamycoplasma hyosynoviae	patients	0.372	0.058	6.387	0.000
17	Halomonas sp. Y2R2	controls	-0.376	0.053	-7.105	0.001
18	Moraxella nonliquefaciens	controls	-0.395	0.066	-5.950	0.001
19	Endozoicomonas montiporae	controls	-0.361	0.053	-6.810	0.001
20	Rhodococcus sp. WAY2	controls	-0.316	0.054	-5.867	0.001
21	Hydrogenophaga sp. NH-16	controls	-0.336	0.054	-6.215	0.001
22	Limosilactobacillus gastricus	patients	0.418	0.071	5.915	0.001
23	Streptomyces sp. BSE6.1	patients	0.400	0.054	7.456	0.001
24	Mahella australiensis	controls	-0.316	0.053	-5.964	0.001
25	Bacillus smithii	patients	0.335	0.053	6.302	0.002
26	Bradyrhizobium sp. CCBAU 53338	patients	0.345	0.052	6.582	0.002
27	Rhizobium sp. X9	patients	0.371	0.053	6.934	0.002
28	Burkholderia sp. MS455	patients	0.382	0.053	7.270	0.002
29	Exiguobacterium sp. MH3	patients	0.373	0.053	7.008	0.002
30	Halomonas sp. N3-2A	controls	-0.328	0.053	-6.178	0.003
31	Candidatus Absconditicoccus praedator	controls	-0.308	0.053	-5.773	0.003
32	Bacillus sp. FJAT-14266	controls	-0.314	0.055	-5.670	0.003
33	Fusarium venenatum	controls	-0.317	0.057	-5.516	0.003
34	Moineauvirus Abc2	controls	-0.351	0.058	-6.025	0.006
35	Burkholderia sp. Bp7605	patients	0.309	0.053	5.836	0.007
36	Hypericibacter adhaerens	controls	-0.329	0.052	-6.270	0.009

37	Dybvigvirus crAssphage cr131_1	patients	0.338	0.057	5.886	0.009
38	Brucella anthropi	controls	-0.372	0.072	-5.200	0.009
39	Mesorhizobium sp. NZP2234	patients	0.310	0.052	5.902	0.016
40	Sulfurimonas lithotrophica	patients	0.309	0.052	5.906	0.018
41	Chlorobium phaeovibrioides	patients	0.278	0.053	5.242	0.019
42	Mycoplasma miroungirhinis	controls	-0.297	0.053	-5.583	0.021
43	Microbacterium sp. SGAir0570	controls	-0.323	0.055	-5.846	0.022
44	Parabacteroides sp. CT06	controls	-0.296	0.059	-5.015	0.023
45	Kiritimatiella glycovorans	controls	-0.267	0.053	-5.050	0.027
46	Clostridioides sp. ES-S-0010-02	patients	0.273	0.053	5.114	0.027
47	Pseudorhodoplanes sinuspersici	patients	0.283	0.052	5.422	0.029
48	Metamycoplasma phocicerebrale	patients	0.297	0.055	5.418	0.035
49	Cobetia sp. 4B	patients	0.289	0.053	5.415	0.042
50	Sedimentisphaera salicampi	patients	0.298	0.052	5.678	0.044
51	Methylobacterium brachiatum	controls	-0.267	0.055	-4.877	0.046
