# Supplementary material for: Multikingdom oral microbiome interactions in early-onset cryptogenic ischemic stroke
Source: ISME Commun. 2024 Jun 20;4(1):ycae088. doi: 10.1093/ismeco/ycae088 (PMC11235082; doi:10.1093/ismeco/ycae088)
Supplement: Supplemental_Material_ycae088_Table_S6 [file supplemental_material_ycae088_table_s6.pdf]

Table S6. List of MetaCyc pathways significantly altered between patients and controls (original *p* <0.05 and *q* < 0.25, Wilcoxon rank-sum test).

**MetaCyc pathway**	**MetaCyc ID**	**Enrich group**	**effect size**	***p-value***	***p*–adj**
8-amino-7-oxononanoate biosynthesis I	PWY-6519	control	-1.64E-06	0.001	0.117
inosine-5'-phosphate biosynthesis II	PWY-6124	patient	3.73E-06	0.002	0.135
tetrapyrrole biosynthesis II (from glycine)	PWY-5189	control	-3.68E-06	0.004	0.193
stachyose degradation	PWY-6527	patient	3.26E-06	0.006	0.240
inosine-5'-phosphate biosynthesis III	PWY-7234	patient	3.75E-06	0.007	0.246
