# Supplementary material for: Multikingdom oral microbiome interactions in early-onset cryptogenic ischemic stroke
Source: ISME Commun. 2024 Jun 20;4(1):ycae088. doi: 10.1093/ismeco/ycae088 (PMC11235082; doi:10.1093/ismeco/ycae088)
Supplement: Supplemental_Material_ycae088_Table_S7 [file supplemental_material_ycae088_table_s7.pdf]

Table S7. The correlation-based network attributes and significant node table from the network analysis for patients with cryptogenic ischemic stroke and controls.

	Patients	Controls						
Vertex	73.000	79.00						
Edge	68.000	70.000						
Average_degree	1.8630	1.7721						
Average_path_length	5.9985	4.7439						
Network_diameter	13.000	11.000						
Clustering_coefficient	0.0000	0.000						
Density	0.0258	0.0227						
Heterogeneity	0.5240	0.6256						
Centralization	0.0435	0.0542						
Modularity	0.8051	0.8003						
**Patients**								
**taxa_roles**	**degree**	**betweenness_ centrality**	**closeness_ centrality**	**eigenvector_ centrality**	**Abundance**	**module**	**z**	**p**
Peripheral nodes	3	95	0.004	0.151	0.546	M3	1.155	0.000
Peripheral nodes	2	12	0.078	0.000	0.144	M5	0.354	0.000
Peripheral nodes	3	14	0.078	0.000	0.636	M5	1.768	0.000
Peripheral nodes	2	48	0.003	0.033	0.108	M6	0.378	0.000
Peripheral nodes	1	0	0.003	0.012	0.134	M2	-1.225	0.000
Peripheral nodes	5	411	0.003	1.000	0.211	M1	2.345	0.000
Peripheral nodes	2	138	0.004	0.268	0.379	M3	0.000	0.000
Peripheral nodes	2	0	0.004	0.498	0.105	M3	0.000	0.000
Peripheral nodes	1	0	0.381	0.000	0.168	M10	-0.577	0.000
Peripheral nodes	1	0	0.003	0.068	0.871	M2	-1.225	0.000
Peripheral nodes	1	0	0.004	0.040	0.574	M4	-0.846	0.000
Peripheral nodes	2	6	0.055	0.000	0.545	M5	0.354	0.000
Peripheral nodes	2	10	0.067	0.000	0.227	M5	0.354	0.000
Peripheral nodes	2	48	0.003	0.331	0.566	M1	0.000	0.000

Peripheral nodes	2	476	0.005	0.476	0.868	M3	0.000	0.000
Peripheral nodes	2	1	0.566	0.000	0.696	M9	1.155	0.000
Peripheral nodes	1	0	0.045	0.000	0.533	M5	-1.061	0.000
Peripheral nodes	1	0	0.371	0.000	0.193	M8	-0.577	0.000
Peripheral nodes	3	176	0.004	0.156	0.819	M2	1.225	0.000
Peripheral nodes	3	183	0.004	0.159	0.200	M6	1.701	0.000
Peripheral nodes	1	0	0.003	0.013	0.956	M6	-0.945	0.000
Peripheral nodes	2	1	0.559	0.000	0.643	M8	1.155	0.000
Peripheral nodes	4	182	0.003	0.761	0.125	M1	1.563	0.000
Peripheral nodes	2	94	0.004	0.067	0.207	M6	0.378	0.000
Peripheral nodes	1	0	0.003	0.058	0.569	M3	-1.155	0.000
Peripheral nodes	1	0	0.003	0.388	0.193	M1	-0.782	0.000
Peripheral nodes	1	0	0.042	0.000	0.132	M5	-1.061	0.000
Peripheral nodes	1	0	0.003	0.004	0.248	M4	-0.846	0.000
Peripheral nodes	1	0	0.367	0.000	0.901	M9	-0.577	0.000
Peripheral nodes	1	0	0.003	0.059	6.306	M3	-1.155	0.000
Peripheral nodes	1	0	0.003	0.389	0.212	M1	-0.782	0.000
Peripheral nodes	2	1	0.581	0.000	0.131	M10	1.155	0.000
Peripheral nodes	1	0	0.374	0.000	0.274	M8	-0.577	0.000
Peripheral nodes	1	0	0.002	0.118	0.354	M1	-0.782	0.000
Peripheral nodes	1	0	0.389	0.000	0.454	M9	-0.577	0.000
Peripheral nodes	2	94	0.003	0.070	2.776	M2	0.000	0.000
Peripheral nodes	2	44	0.003	0.248	0.958	M1	0.000	0.000
Peripheral nodes	2	6	0.003	0.228	1.401	M1	0.000	0.000
Peripheral nodes	3	140	0.003	0.022	7.137	M4	1.410	0.000
Peripheral nodes	2	6	0.003	0.112	0.207	M2	0.000	0.000
Peripheral nodes	1	0	0.003	0.048	1.232	M7	-0.866	0.000
Peripheral nodes	1	0	0.004	0.084	2.609	M2	-1.225	0.000
Peripheral nodes	1	0	0.003	0.020	4.193	M7	-0.866	0.000
Peripheral nodes	1	0	0.056	0.000	1.273	M5	-1.061	0.000

Peripheral nodes	2	48	0.003	0.053	0.101	M7	0.866	0.000
Peripheral nodes	1	0	0.004	0.061	0.416	M6	-0.945	0.000
Peripheral nodes	2	48	0.003	0.010	1.029	M4	0.282	0.000
Peripheral nodes	2	84	0.003	0.457	0.538	M1	0.000	0.000
Peripheral nodes	2	8	0.003	0.368	0.288	M1	0.000	0.000
Peripheral nodes	2	180	0.004	0.043	1.040	M4	0.282	0.000
Peripheral nodes	1	0	0.003	0.287	0.368	M1	-0.782	0.000
Peripheral nodes	2	6	0.059	0.000	0.224	M5	0.354	0.000
Peripheral nodes	3	263	0.004	0.099	0.139	M4	1.410	0.000
Peripheral nodes	1	0	0.003	0.008	0.209	M4	-0.846	0.000
Peripheral nodes	1	0	0.394	0.000	0.293	M10	-0.577	0.000
Peripheral nodes	1	0	0.004	0.281	0.120	M3	-1.155	0.000
Peripheral nodes	2	48	0.003	0.032	0.393	M2	0.000	0.000
Peripheral nodes	2	4	0.003	0.100	0.136	M2	0.000	0.000

Controls

**taxa_roles**	**degree**	**betweenness_ centrality**	**closeness_ centrality**	**eigenvector_ centrality**	**Abundance**	**module**	**z**	**p**
Peripheral nodes	2	50	0.004	0.034	0.194	M6	0.645	0.000
Peripheral nodes	2	1	0.587	0.000	1.367	M9	1.155	0.000
Peripheral nodes	2	50	0.003	0.017	0.108	M1	0.242	0.000
Peripheral nodes	1	0	0.005	0.263	0.106	M2	-0.846	0.000
Peripheral nodes	4	193	0.004	0.180	0.338	M7	1.927	0.000
Peripheral nodes	1	0	0.005	0.124	0.239	M1	-1.090	0.000
Peripheral nodes	2	98	0.004	0.095	1.054	M6	0.645	0.000
Peripheral nodes	2	50	0.005	0.355	1.140	M4	0.300	0.000
Peripheral nodes	1	0	0.003	0.017	0.187	M8	-0.645	0.000
Peripheral nodes	1	0	0.061	0.000	0.537	M5	-0.945	0.000
Peripheral nodes	2	1	0.584	0.000	0.548	M10	1.155	0.000
Peripheral nodes	1	0	0.003	0.012	0.924	M6	-1.291	0.000
Peripheral nodes	2	5	0.080	0.000	0.260	M5	0.378	0.000

Peripheral nodes	2	50	0.004	0.129	0.110	M2	0.282	0.000
Peripheral nodes	3	279	0.004	0.104	0.507	M1	1.574	0.000
Peripheral nodes	1	0	0.003	0.004	0.526	M1	-1.090	0.000
Peripheral nodes	2	50	0.003	0.014	0.794	M1	0.242	0.000
Peripheral nodes	3	99	0.005	0.451	0.582	M4	1.352	0.000
Peripheral nodes	1	0	0.004	0.072	0.787	M3	-0.935	0.000
Peripheral nodes	1	0	0.004	0.144	1.043	M4	-0.751	0.000
Peripheral nodes	1	0	0.004	0.050	0.111	M3	-0.935	0.000
Peripheral nodes	1	0	0.005	0.239	0.402	M6	-1.291	0.000
Peripheral nodes	3	99	0.005	0.324	0.154	M2	1.410	0.000
Peripheral nodes	3	11	0.111	0.000	0.175	M5	1.701	0.000
Peripheral nodes	1	0	0.382	0.000	0.611	M10	-0.577	0.000
Peripheral nodes	2	98	0.004	0.036	0.305	M1	0.242	0.000
Peripheral nodes	1	0	0.003	0.005	1.009	M1	-1.090	0.000
Peripheral nodes	1	0	0.388	0.000	6.882	M9	-0.577	0.000
Peripheral nodes	1	0	0.058	0.000	0.165	M5	-0.945	0.000
Peripheral nodes	1	0	0.004	0.061	0.135	M7	-0.550	0.000
Peripheral nodes	1	0	0.394	0.000	0.305	M9	-0.577	0.000
Peripheral nodes	1	0	0.004	0.070	0.312	M1	-1.090	0.000
Peripheral nodes	2	98	0.004	0.040	5.848	M1	0.242	0.000
Peripheral nodes	1	0	0.004	0.056	1.414	M7	-0.550	0.000
Peripheral nodes	1	0	0.004	0.159	1.091	M4	-0.751	0.000
Peripheral nodes	2	36	0.005	0.180	0.195	M3	0.000	0.000
Peripheral nodes	4	99	0.005	0.240	2.113	M3	1.871	0.000
Peripheral nodes	3	99	0.003	0.048	4.288	M8	1.291	0.000
Peripheral nodes	1	0	0.003	0.019	1.330	M7	-0.550	0.000
Peripheral nodes	3	191	0.004	0.122	0.872	M8	1.291	0.000
Peripheral nodes	1	0	0.004	0.111	1.035	M4	-0.751	0.000
Peripheral nodes	2	8	0.101	0.000	0.205	M5	0.378	0.000
Peripheral nodes	2	50	0.004	0.059	0.337	M7	0.275	0.000

Peripheral nodes	1	0	0.074	0.000	0.108	M5	-0.945	0.000
Peripheral nodes	2	144	0.005	0.287	7.289	M6	0.645	0.000
Peripheral nodes	1	0	0.004	0.109	0.200	M2	-0.846	0.000
Peripheral nodes	1	0	0.003	0.040	0.104	M8	-0.645	0.000
Peripheral nodes	1	0	0.004	0.111	0.200	M2	-0.846	0.000
Peripheral nodes	1	0	0.398	0.000	0.177	M10	-0.577	0.000
Peripheral nodes	2	5	0.086	0.000	0.228	M5	0.378	0.000
Peripheral nodes	3	46	0.004	0.191	3.902	M3	0.935	0.000
Peripheral nodes	1	0	0.003	0.045	0.289	M2	-0.846	0.000
Peripheral nodes	1	0	0.003	0.015	0.155	M8	-0.645	0.000
Peripheral nodes	1	0	0.004	0.079	0.148	M3	-0.935	0.000
Peripheral nodes	1	0	0.005	0.326	0.587	M4	-0.751	0.000
Peripheral nodes	2	98	0.005	0.316	0.364	M2	0.282	0.000
